# Associations between Mental Health and Ebola-Related Health Behaviors: A Regionally Representative Cross-sectional Survey in Post-conflict Sierra Leone

**DOI:** 10.1371/journal.pmed.1002073

**Published:** 2016-08-09

**Authors:** Theresa S. Betancourt, Robert T. Brennan, Patrick Vinck, Tyler J. VanderWeele, Dayo Spencer-Walters, Joshua Jeong, Adeyinka M. Akinsulure-Smith, Phuong Pham

**Affiliations:** 1 Department of Global Health and Population, Harvard T. H. Chan School of Public Health, Boston, Massachusetts, United States of America; 2 Department of Emergency Medicine, Harvard Medical School and Brigham and Women’s Hospital, Boston, Massachusetts, United States of America; 3 Department of Epidemiology, Harvard T. H. Chan School of Public Health, Boston, Massachusetts, United States of America; 4 Department of Biostatistics, Harvard T. H. Chan School of Public Health, Boston, Massachusetts, United States of America; 5 Department of Psychology, City College of New York, New York, New York, United States of America; Western Sydney University, AUSTRALIA

## Abstract

**Background:**

Little attention has been paid to potential relationships between mental health, trauma, and personal exposures to Ebola virus disease (EVD) and health behaviors in post-conflict West Africa. We tested a conceptual model linking mental health and trauma to EVD risk behaviors and EVD prevention behaviors.

**Methods and Findings:**

Using survey data from a representative sample in the Western Urban and Western Rural districts of Sierra Leone, this study examines associations between war exposures, post-traumatic stress disorder (PTSD) symptoms, depression, anxiety, and personal EVD exposure (e.g., having family members or friends diagnosed with EVD) and EVD-related health behaviors among 1,008 adults (98% response rate) from 63 census enumeration areas of the Western Rural and Western Urban districts randomly sampled at the height of the EVD epidemic (January–April 2015). Primary outcomes were EVD risk behaviors (14 items, Cronbach’s α = 0.84) and EVD prevention behaviors (16 items, Cronbach’s α = 0.88). Main predictors comprised war exposures (8 items, Cronbach’s α = 0.85), anxiety (10 items, Cronbach’s α = 0.93), depression (15 items, Cronbach’s α = 0.91), and PTSD symptoms (16 items, Cronbach’s α = 0.93). Data were analyzed using two-level, population-weighted hierarchical linear models with 20 multiply imputed datasets. EVD risk behaviors were associated with intensity of depression symptoms (*b* = 0.05; 95% CI 0.00, 0.10; *p =* 0.037), PTSD symptoms (*b* = 0.10; 95% CI 0.03, 0.17; *p =* 0.008), having a friend diagnosed with EVD (*b* = −0.04; 95% CI −0.08, −0.00; *p =* 0.036), and war exposures (*b* = −0.09; 95% CI −0.17, −0.02; *p =* 0.013). EVD prevention behaviors were associated with higher anxiety (*b* = 0.23; 95% CI 0.06, 0.40; *p =* 0.008), having a friend diagnosed with EVD (*b* = 0.15; 95% CI 0.04, 0.27; *p =* 0.011), and higher levels of war exposure (*b* = 0.45; 95% CI 0.16, 0.74; *p =* 0.003), independent of mental health. PTSD symptoms were associated with lower levels of EVD prevention behavior (*b* = −0.24; 95% CI −0.43, −0.06; *p =* 0.009).

**Conclusions:**

In post-conflict settings, past war trauma and mental health problems are associated with health behaviors related to combatting EVD. The associations between war trauma and both EVD risk behaviors and EVD prevention behaviors may be mediated through two key mental health variables: depression and PTSD symptoms. Considering the role of mental health in the prevention of disease transmission may help fight continuing and future Ebola outbreaks in post-conflict Sierra Leone. This sample is specific to Freetown and the Western Area and may not be representative of all of Sierra Leone. In addition, our main outcomes as well as personal EVD exposure, war exposures, and mental health predictors rely on self-report, and therefore raise the possibility of common methods bias. However, the findings of this study may be relevant for understanding dynamics related to EVD and mental health in other major capital cities in the EVD-affected countries of West Africa.

## Abstract 2

Theresa Betancourt and colleagues examine the associations between mental health and Ebola-related health behaviors in post-conflict Sierra Leone.

## Author Summary

**Why Was This Study Done?:**

- Little attention has been paid to how mental health, trauma, personal exposures to Ebola virus disease (EVD) relate to health behaviors in post-conflict West Africa.
- This study was done to test the links between mental health and trauma and health behaviors related to EVD—behaviors that increase the risk of spreading EVD and behaviors that help to prevent the spread of EVD.

**What Did the Researchers Do and Find?:**

- At the height of the EVD epidemic (January–April 2015), 1,008 adults selected at random from 63 census enumeration areas in the Western Rural and Western Urban districts of Sierra Leone were surveyed to examine the relationships between war exposures, post-traumatic stress disorder (PTSD) symptoms, depression, anxiety, personal exposure to EVD, and EVD-related health behaviors.
- Individuals reporting greater intensity of depression symptoms and higher rates of PTSD symptoms also reported higher rates of behaviors that increase the risk of spreading EVD, while individuals reporting previous exposure to war or having a friend diagnosed with EVD reported lower rates of such behaviors.
- Individuals reporting higher levels of anxiety, having a friend diagnosed with EVD, and greater previous exposure to war (taking into account their mental health status) reported increased rates of behaviors that help to prevent the spread of EVD, while individuals reporting higher rates of PTSD reported lower rates of such behaviors.

**What Do These Findings Mean?:**

- These findings suggest that in areas with a recent history of war or conflict, past war trauma and certain mental health problems are linked with health behaviors that are important in stopping the spread of EVD.
- Considering the role of mental health, in particular depression and PTSD, in preventing the spread of disease may help fight ongoing and future Ebola outbreaks in Sierra Leone.
- While this study is specific to the Western Area of Sierra Leone, its implications are important for countries in West Africa affected by conflict and EVD.

## Introduction

In many post-conflict settings, years of protracted violence have left the populace exposed to war trauma and loss, which contribute to unmet mental health needs. A history of conflict in West Africa has destroyed health systems, undermined community relations, and reduced trust in state institutions, increasing risks for health crises and social unrest [1]. In Sierra Leone, the health system was decimated over the course of the 11-y civil war (1991–2002). An estimated 50,000 persons were killed, and over 20,000 children and adolescents were involved with armed groups [2]. In Sierra Leone today, health services remain underdeveloped, of poor quality, and fraught with barriers to accessing care [3]. Mental health and social services are nearly nonexistent [4].

Direct and indirect effects of war likely contributed to the ability of Ebola virus disease (EVD) to ravage the country in 2014–2015. This EVD outbreak in Sierra Leone is reported to have resulted in 8,704 confirmed cases (13,823 confirmed, probable, and suspected cases) and 3,589 confirmed deaths (3,955 confirmed, probable, and suspected deaths) [5] and laid bare the inadequacies of the health system, which was unable to manage the crisis at its peak. To prevent future EVD outbreaks in Sierra Leone, it is important to understand how unmet mental health and social service needs influence both EVD risk and health behaviors critical for EVD prevention.

**Fig 1:**
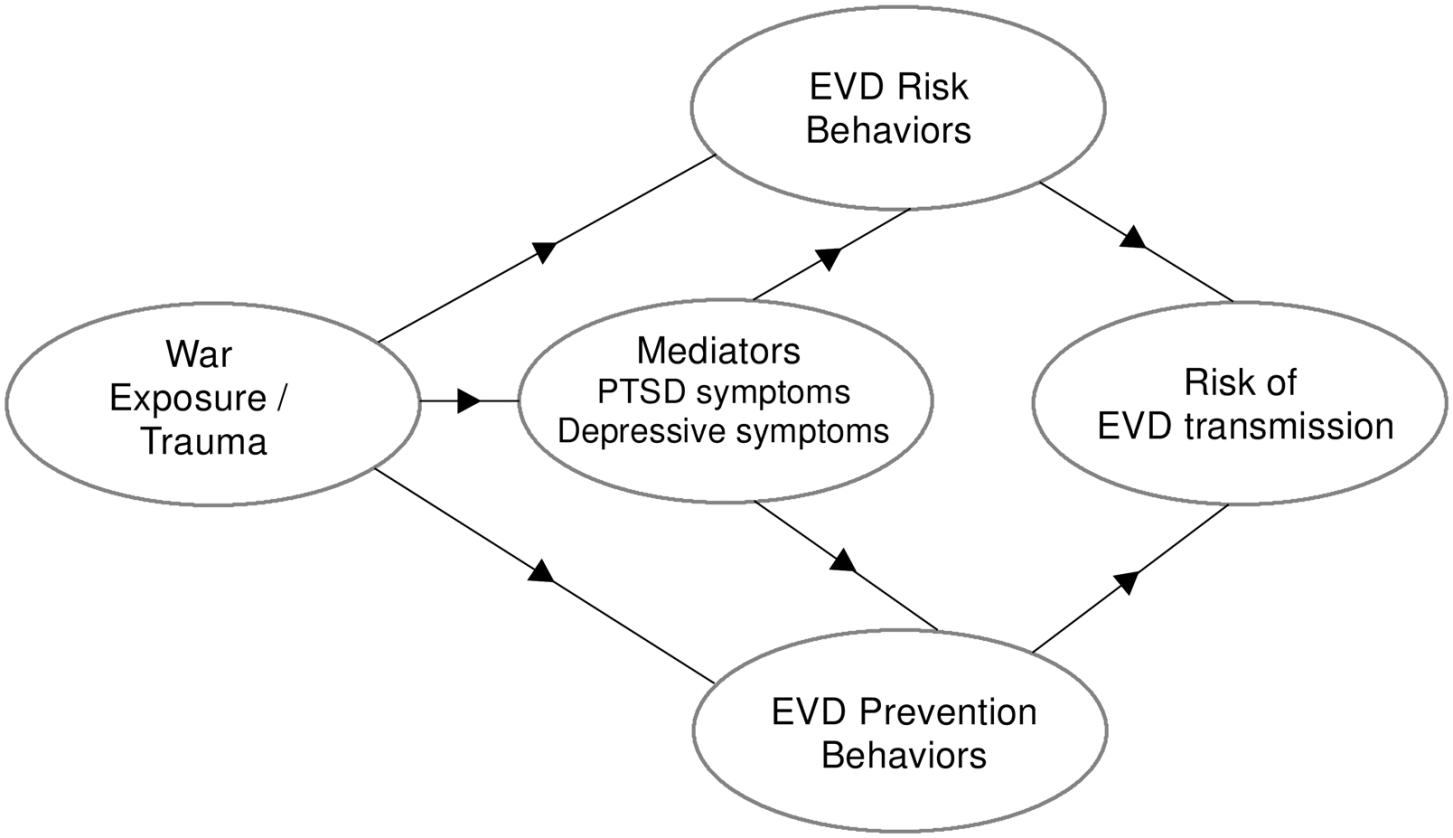
Conceptual model for war exposures, mental health, and EVD-related behaviors.

Research has documented associations between war exposures, mental health problems, and impairments in functioning among survivors of trauma including war [6–11]. However, in the context of EVD in West Africa, associations between past trauma, ongoing mental health difficulties, and health behaviors remain unexplored. Drawing from theories examining adaptive coping to life stressors as well as research investigating disease risk behaviors in the context of HIV/AIDS [12–15], Fig 1 sets out a conceptual model whereby exposure to past trauma may affect both EVD risk behaviors and the uptake of prevention messaging and EVD prevention/health-promoting behaviors, and where this effect may be mediated by related mental health difficulties. Our hypotheses are grounded in previous research on mental health and the dynamics of infectious disease prevention. For instance, poor adherence to treatment for HIV/AIDS has been linked to depression as well as post-traumatic stress disorder (PTSD) and other anxiety disorders [16–20]. Similarly, we hypothesized that in the context of EVD, past trauma and depression would relate to poor problem-solving in the context of EVD. We theorized that PTSD and depression might contribute to hopelessness and a foreshortened sense of the future, which might increase the likelihood of risk behavior. Understanding the relationship between past war exposures, mental health, and EVD risk and EVD prevention behaviors might provide critical knowledge for EVD prevention in Sierra Leone and other post-conflict settings.

## Methods

Institutional review board approval was obtained from the Harvard T. H. Chan School of Public Health (Protocol #15145, Approval #17) and the Sierra Leone Ministry of Health and Sanitation Ethics and Scientific Review Committee. With institutional review board approval, consent was obtained orally with a witness due to low literacy among participants. A local community advisory board, comprising community members and healthcare professionals, reviewed and advised the research.

**Fig 2:**
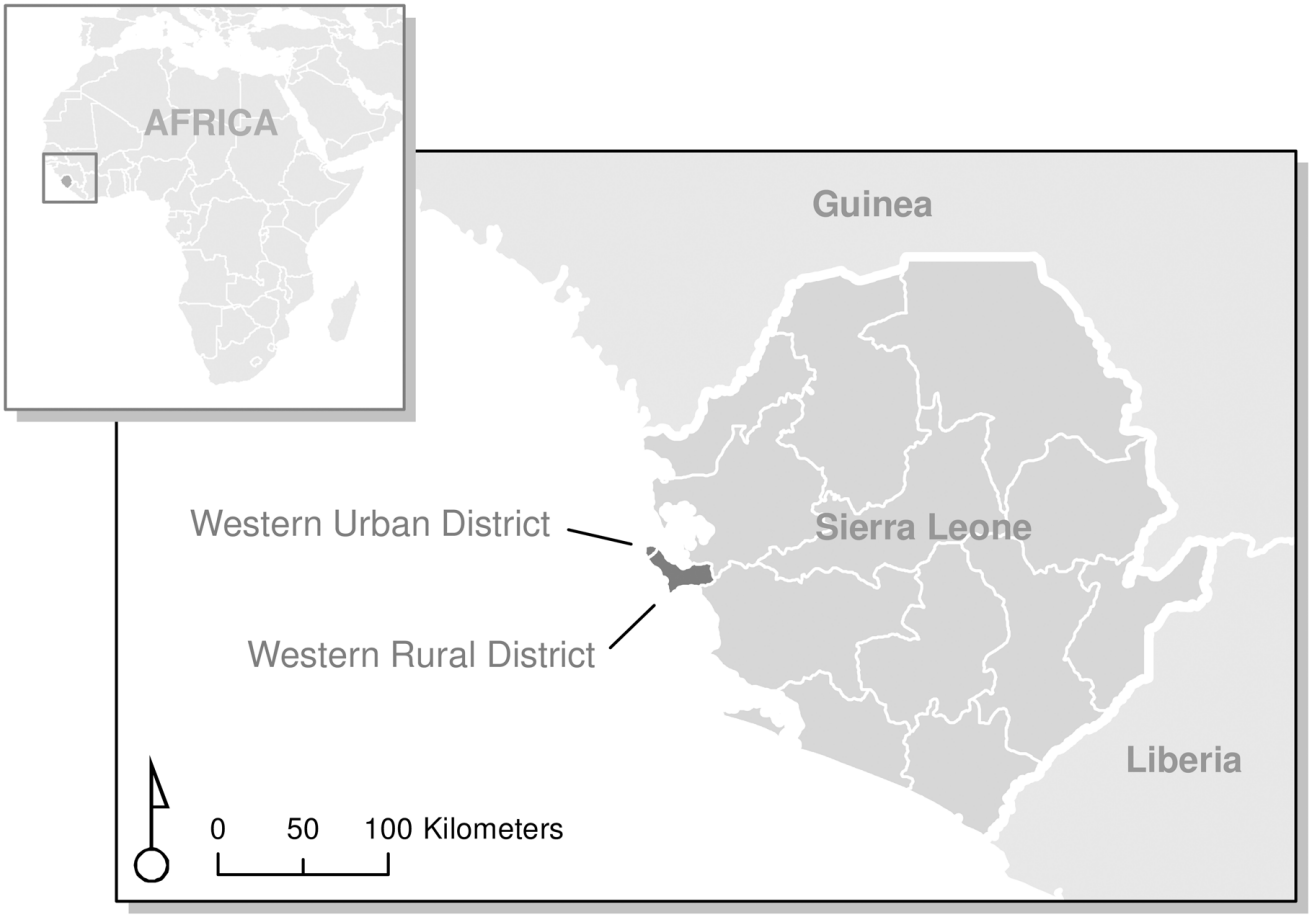
Sampled districts (Western Urban and Western Rural) in Sierra Leone. Map by the authors, based on 2012 global administrative areas in GADM, version 2.0 (http://www.gadm.org).

This survey was conducted January–April 2015 in the Western Urban (including the capital, Freetown) and the Western Rural districts of Sierra Leone (Fig 2). These districts represent diversity in ethnic composition and degrees of war exposure and were the epicenter of EVD cases during the 2014–2015 outbreak, together accounting for over 40% of confirmed cases.

### Sample

**Fig 3:**
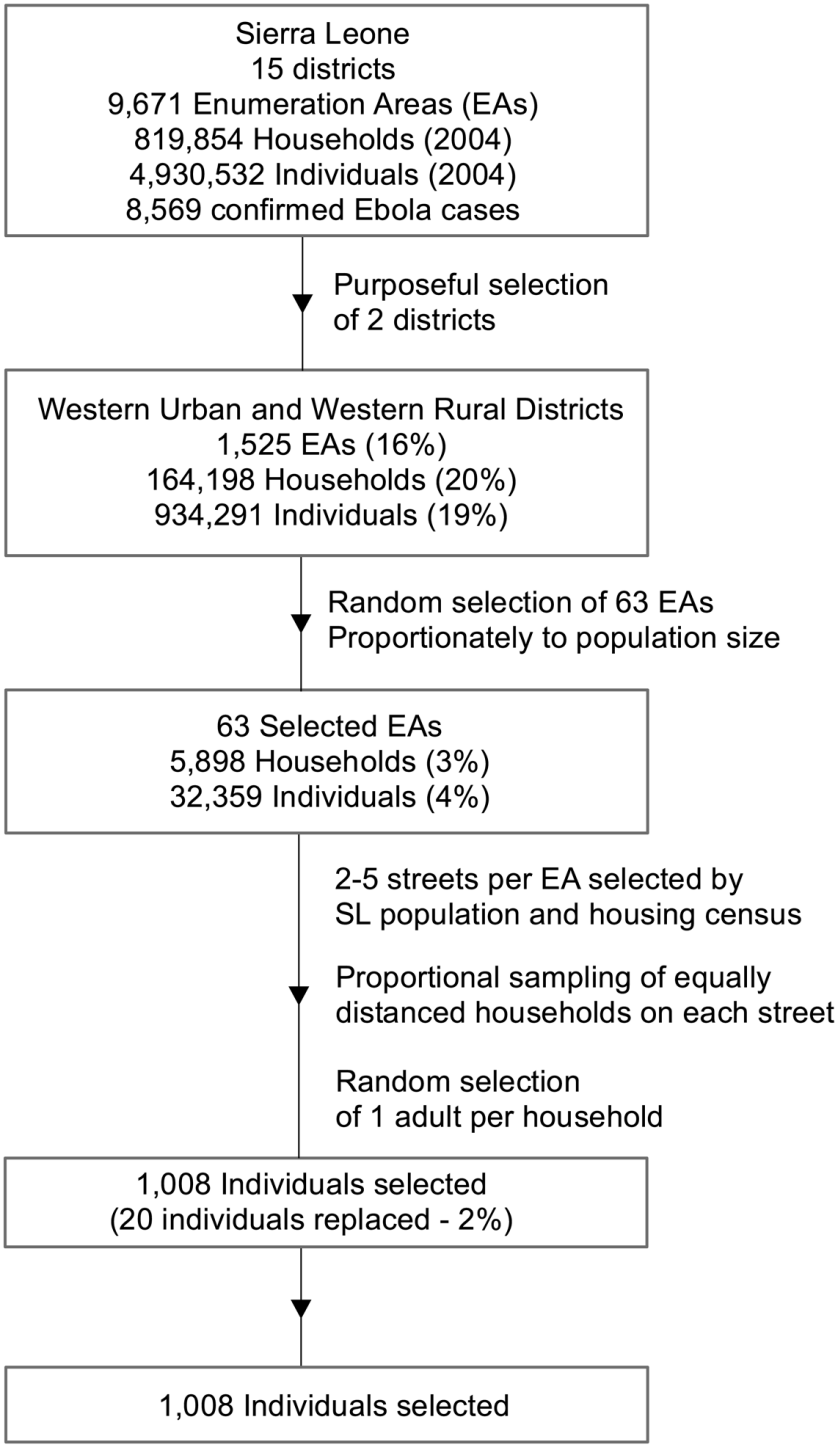
Sampling procedure. SL, Sierra Leone.

The sampling population included adults 18 y and older living in the Western Urban and Western Rural districts, including Sierra Leone’s capital. Participants were selected using multistage cluster sampling. Census enumeration areas (EAs) were the primary sampling unit (see Fig 3). A list of EAs for the two districts and maps defining the EA boundaries were obtained from Statistics Sierra Leone [21]. Sixty-three EAs were randomly sampled from the list. Sixteen households within each EA were selected using random geographic sampling techniques. For each EA, a map was provided by Statistics Sierra Leone with a specified number of streets (2–5) indicated encompassing the EAs for the Sierra Leone 2004 Population and Housing Census. On those streets, the interviewers then selected a proportional sample of equally distanced households (with “household” defined as persons residing together). Each household was approached, and among adults who happened to be available on first contact, one was chosen at random from the household by alphabetizing first names in ascending order and choosing the first one. When a selected individual was unable or refused to participate, another individual within the selected household was randomly selected. After three attempts over the course of one day, if no household member was available, another household was selected using the same geographic randomization techniques.

### Study Measures

The EVD risk behaviors outcome (Cronbach’s α = 0.84) was measured by 14 three-point items assessing likelihood (0 = very unlikely, 1 = somewhat likely, 2 = very likely) of engaging in certain behaviors if a family member or oneself experienced EVD-like symptoms (fever, fatigue, malaise and weakness, reddened eyes, joint and muscle pain, headache, nausea and vomiting). Such risk behaviors included waiting to see if the symptoms would go away, trying to treat the symptoms at home, and trying treatments such as hot salt water baths (see S1 Table for full list of risk behavior items). The EVD prevention behaviors outcome comprised a set of 16 items (α = 0.88) scored on a four-point frequency scale (0 = never, 1 = sometimes, 2 = often, 3 = always); EVD prevention behaviors included frequent hand washing, seeking EVD prevention information from a healthcare worker, and avoidance of large public events, public transport, and touching dead bodies, consistent with prevention messaging being used in Sierra Leone at the time of the study (see S2 Table for full list of prevention behavior items).

Independent variables (predictors) included an adapted version of the War Trauma Questionnaire, comprising 16 items, used in Liberia and the Democratic Republic of the Congo [22,23] to assess conflict-related experiences (eight items) and witnessing experiences (four items). Based on frequency of occurrence in the sample, item–total correlations, and internal consistency estimates, the 16 war exposure items in the original questionnaire were reduced to eight items addressing five victimization experiences (being forced to flee, having one’s home destroyed, being physically beaten, being forced to work or commit violence, being threatened with death) and three witnessing experiences (looting and destruction, beating or torture, killing of a household member). These were coded as 1 = occurrence or 0 = no occurrence and combined into one scale (α = 0.85). Anxiety and depression were assessed using a Krio (the lingua franca of Sierra Leone) adaptation of the Hopkins Symptom Checklist-25 (HSCL-25), scored on past week symptom intensity (1 = not at all, 2 = a little, 3 = quite a bit, 4 = extremely), previously adapted for Sierra Leone [24–26]. Both the depression (α = 0.91) and anxiety (α = 0.93) subscales had excellent internal consistency. To assess symptoms of PTSD, participants responded to 16 items (0 = no, 1 = yes) about the most distressing event they had ever experienced or witnessed, using the PTSD Symptom Scale–Interview adapted for use in Liberia (see S3 Table; α = 0.93) [27]. To assess personal EVD exposure, participants were asked five questions to determine whether a household member, a family member whom they did not live with, a friend, a neighbor, or someone in their community had been diagnosed with EVD over the past 12 mo (0 = no, 1 = yes).

Mental health scales used had been previously validated or used in Sierra Leone, Liberia, or other conflict-affected countries in sub-Saharan Africa [24–29]. All measures new to this setting were reviewed by local collaborators for face validity, examined item by item for local comprehension, and forward- and backward-translated following a standard protocol [30–32].

All continuous scales are averaged; thus, they are interpreted in terms of their original response scale.

### Procedures

Local research assistants (eight women and seven men) were trained to administer the survey using Android tablets running Kobo Toolbox digital data collection software [33]. All research assistants received a 3-d training on survey administration and research ethics. Interviewers were assigned to same-sex participants. Interviews were conducted one-on-one in an outdoor setting (due to ongoing EVD outbreak) that provided privacy and confidentiality. Participants were given contact information to use if they had any questions and were offered a small gift of foodstuffs (value US$2) for agreeing to be interviewed. Risk of harm cases were referred to local mental health or social services for follow-up care (two cases were identified and referred).

### Data Analysis Plan

All descriptive statistical analyses were conducted in STATA 13.0 SE [34]. Using HLM 7.0 [35], we estimated two-level hierarchical linear models (also called “multilevel” or “mixed” models) to accommodate the clustering of data into EAs and to prevent bias in estimation of standard errors. Because this was an equal allocation sampling design based on 16 households per EA, weights were applied based on the population of each EA to allow generalization to the Western Urban and Western Rural districts from which the EAs were sampled. Households in two EAs were undersampled because the EAs had been improperly identified (clerical error), so the remaining households were up-weighted so that those EAs would be properly represented in the sample. Furthermore, the households that had been misidentified were placed in their proper EAs, resulting in oversampling of those EAs; households in these EAs were, therefore, down-weighted to prevent overrepresentation in the weighted sample. Robust standard errors were used for all model interpretation.

In accordance with our conceptual model, multilevel models predicting EVD prevention behaviors and EVD risk behaviors were fit, containing the three mental health variables—anxiety, depression, and PTSD symptoms—as well as war exposures and EVD diagnosis of people in their family or community (details described above). To examine PTSD symptoms, anxiety, and depression as mediators of EVD risk and prevention behaviors, and to estimate the direct and indirect effects of war exposures, we fit multilevel models in which each of the outcomes (EVD prevention behaviors and EVD risk behaviors) was regressed on war exposures (with measures of personal exposure to EVD and anxiety as controls) and each of the mental health outcomes, and also models in which two of the mental health variables (PTSD symptoms and depression symptoms) were regressed on war exposures [36]. Because the temporal sequencing of EVD-related health behaviors and anxiety was less clear than that of the other two mental health variables, anxiety was not considered as a mediator. Mediation analyses assumed that the effect of the exposure and mediator on the outcome were unconfounded.

In order to make use of all available data (*n =* 1,008) and avoid possible bias associated with listwise missing value deletion, 20 multiply imputed datasets were created for the analyses, using all variables included in the analyses plus additional demographic variables (participant age, sex, marital status, education, and household wealth, based on household land and asset ownership) and participant report of daily hardships for the imputations. The number of missing values for a given variable can be determined by comparing the *n* for a given variable in Tables 1 and 2 (e.g., 979 for depression symptoms score) to 1,008, the total number of participants interviewed.

**Table 1:** Unweighted and weighted sociodemographic profile of participants.

Characteristic	*N*	Unweighted Percent (95% CI)	Weighted Percent (95% CI)
**Sex (*n =* 1,008)**			
Male	503	49.9	49.2
Female	505	50.1	50.8
**Age, mean, y (*n =* 1,008)**	1,008	33.8 (32.9, 34.6)	34.2 (33.2, 35.2)
**Number of assets (of 9), mean (*n =* 1,008)**	1,008	3.6 (3.5, 3.8)	3.5 (3.2, 3.9)
**Marital status (*n =* 1,008)**			
Currently married	424	42.1 (39.0, 45.1)	42.7 (38.8, 46.7)
Widowed	49	4.9 (3.7, 6.4)	6.0 (4.4, 8.1)
Currently living with a partner but not married	21	2.1 (1.4, 3.2)	2.1 (1.2, 3.5)
Never married/never lived with a partner	455	45.1 (42.1, 48.2)	43.2 (39.4, 47.1)
Other^a^	59	5.9 (4.6, 7.5)	6.0 (4.5, 8.1)
**Education (*n =* 1,006)**			
None	163	16.2 (14.0, 18.6)	18.5 (14.6, 23.2)
Some primary	48	4.8 (3.6, 6.3)	4.7 (3.5, 6.3)
Completed primary	17	1.7 (1.1, 2.7)	1.7 (0.9, 3.1)
Some secondary	380	37.8 (34.8, 40.8)	37.9 (34.0, 41.9)
Completed secondary	239	23.8 (21.2, 26.5)	22.0 (18.7, 25.6)
Some post-secondary	86	8.5 (7.0, 10.4)	8.2 (6.5, 10.4)
Completed post-secondary	73	7.3 (5.8, 9.0)	7.0 (5.5, 9.0)
**Ethnicity (*n =* 1,008)**			
Krio	77	7.6 (6.1, 9.5)	6.6 (4.9, 8.9)
Mende	161	16.0 (13.8, 18.4)	15.6 (12.7, 19.2)
Temne	368	36.5 (33.6, 39.5)	40.1 (34.3, 46.1)
Loko	45	4.5 (3.3, 5.9)	4.3 (3.2, 5.9)
Mandingo	45	4.5 (3.3, 5.9)	4.3 (3.0, 6.2)
Kono	20	2.0 (1.3, 3.1)	1.8 (0.9, 3.3)
Soso	31	3.1 (2.2, 4.3)	2.9 (1.9, 4.2)
Fulah	74	7.3 (5.9, 9.1)	6.3 (4.6, 8.6)
Limba	134	13.3 (11.3, 15.5)	13.6 (10.6, 17.2)
Other^b^	53	5.3 (4.0, 6.8)	4.5 (3.0, 6.7)
**Language (*n =* 1,008)**			
Krio	882	87.5 (85.3, 89.4)	88.4 (85.3, 91.0)
Mende	20	2.0 (1.3, 3.1)	1.7 (1.1, 2.8)
Temne	64	6.3 (5.0, 8.0)	5.8 (4.0, 8.3)
Fulah	17	1.7 (1.1, 2.7)	1.8 (1.1, 2.9)
Limba	10	1.0 (0.5, 1.8)	1.2 (0.6, 2.5)
Other^c^	15	1.5 (0.9, 2.5)	1.1 (0.4, 2.5)
**Household owns land (*n =* 1,006)**			
No	801	79.6 (77.0, 82.0)	77.9 (73.3, 81.8)
Yes	205	20.4 (18.0, 23.0)	22.1 (18.2, 26.7)

Survey results are representative of the adult household-based population of the Western Urban and Western Rural districts of Sierra Leone between January and April 2015.

^a^There were 15 who had lived with a partner but were now single, 14 who were engaged, nine who had been separated but were now married, seven who were divorced, and 14 other.

^b^There were eight Kissi, five Valunka, two Kru, one Marankais, and 37 other.

^c^There were two Kono, one English, and 12 other.

**Table 2:** Unweighted and weighted war exposures, mental health symptoms, personal exposure to EVD, and EVD-related health behavior outcomes.

Characteristic	*N*	Unweighted Percent (95% CI)	Weighted Percent (95% CI)
**War exposures (*n =* 1,008)**			
Any war exposure	295	29.3 (26.5, 32.2)	29.7 (24.2, 35.8)
Forced to flee	211	20.9 (18.5, 23.6)	20.8 (16.1, 26.4)
Home/living property destroyed	145	14.4 (12.3, 16.7)	14.7 (11.5, 18.7)
Physically beaten by armed individuals	18	1.8 (1.1, 2.8)	2.1 (1.1, 4.0)
Forced to work or commit violence	18	1.8 (1.1, 2.8)	1.9 (1.0, 3.3)
Threatened with death	61	6.1 (4.7, 7.7)	7.3 (5.0, 10.6)
Witnessed looting/destruction of homes and goods	201	19.9 (17.6, 22.5)	20.3 (16.1, 25.3)
Witnessed beating or torture	126	12.5 (10.6, 14.7)	12.9 (9.7, 16.9)
Witnessed killing of a household member by an armed group	89	8.8 (7.2, 10.7)	8.5 (6.2, 11.6)
War exposures, mean	1,008	0.86 (0.76, 0.96)	0.89 (0.69, 1.08)
**Mental health**			
Depression score, mean^a^	979	1.38 (1.35, 1.42)	1.38 (1.30, 1.46)
Anxiety score, mean^a^	979	1.29 (1.26, 1.33)	1.29 (1.21, 1.37)
Symptoms of PTSD (*n =* 563)	58	10.3 (8.0, 13.1)	11.3 (7.7, 16.3)
Daily hardships	938	0.12 (0.09, 0.16)	0.13 (0.10, 0.17)
**Personal exposure to EVD**			
Someone in the community (*n =* 1,001)	413	41.3 (38.2, 44.3)	44.7 (35.6, 54.3)
Neighbor (*n =* 1,003)	175	17.4 (15.2, 19.9)	20.5 (14.3, 28.4)
Friend (*n =* 1,002)	92	9.2 (7.5, 11.1)	11.0 (6.8, 17.3)
Family member not in the household (*n =* 1,001)	87	8.7 (7.1, 10.6)	11.1 (7.2, 16.6)
Household member (*n =* 1,001)	53	5.3 (4.1, 6.9)	6.9 (3.8, 12.3)
**EVD-related health behaviors**			
EVD risk behaviors, mean	1,007	0.12 (0.11, 0.14)	0.12 (0.09, 0.15)
EVD prevention behaviors, mean	1,008	1.35 (1.31, 1.39)	1.37 (1.29, 1.44)

Survey results are representative of the adult household-based population of the Western Urban and Western Rural districts of Sierra Leone between January and April 2015.

^a^Cutoff score was 1.75, as measured by the HSCL-25.

## Results

Population-weighted estimates for the adult household-based population living in the Western Urban and Western Rural districts of Sierra Leone are presented in Table 1. The sample comprised 505 women (50.8%) and 503 men (49.2%). The mean age of participants was 34.2 y (95% CI 33.2, 35.2 y) and median age was 30 y, ranging from 18 to 84 y. Over 42% (42.7%; 95% CI 38.8%, 46.7%) of the participants reported being in a marital relationship or a partnership; an equal proportion (43.2%; 95% CI 39.4%, 47.1%) reported never being married or living with a partner. About a fifth of the participants reported no formal education (18.5%; 95% CI 14.6%, 23.2%) or incomplete primary schooling (4.7%; 95% CI 3.5%, 6.3%). The main ethnic groups were Temne (40.1%; 95% CI 34.3%, 46.1%), Mende (15.6%; 95% CI 12.7%, 19.2%), and Limba (13.6%; 95% CI 10.6%, 17.2%), and a majority of the participants (88.4%; 95% CI 85.3%, 91.0%) spoke Krio at home. Less than a quarter of participant households (22.1%; 95% CI 18.2%, 26.7%) owned their land, and out of a list of nine common assets, a mean of 3.5 (95% CI, 3.2, 3.9) assets was owned.

Thirty percent of participants (29.7%; 95% CI 24.2%, 35.8%) were exposed to at least one war-related event (Table 2), and the average number of war exposures was 0.88 (95% CI 0.69, 1.08). Of note, 20.8% (95% CI 16.1%, 26.4%) of the sample reported being forced to flee during the war, and 14.7% (95% CI 11.5%, 18.7%) had seen their homes/property destroyed. Just over 2% (2.1%; 95% CI 1.1%, 4.0%) of participants reported being beaten during the war, and 7.3% (95% CI 5.0%, 10.6%) of the sample had been threatened with death. Twenty percent of the sample (20.3%; 95% CI 16.1%, 25.3%) had witnessed looting/destruction of their home or belongings, 12.9% (95% CI 9.7%, 16.9%) had witnessed beating or torture of others, and 8.5% (95% CI 6.2%, 11.6%) had witnessed the killing of a household member by an armed group.

Levels of mental health problems were noteworthy. On the HSCL-25, the mean score was 1.38 (95% CI 1.30, 1.46) for the depression section and 1.29 (95% CI 1.21, 1.37) for the anxiety section. On the PTSD Symptom Scale, among those individuals who endorsed a traumatic war-related event or who chose to discuss a troubling event without describing it (*n =* 563), an estimated weighted prevalence in this sample for meeting the criteria of likely PTSD was 11.3% (95% CI 7.7%, 16.3%).

Close to half the participants reported having EVD cases in their community (44.7%; 95% CI 35.6%, 54.3%), and one in five reported cases among neighbors (20.5%; 95% CI 14.3%, 28.4%). Fewer reported cases among friends (11.0%; 95% CI 6.8%, 17.3%), family members outside the household (11.1%; 95% CI 7.2%, 16.6%), and household members (6.9%; 95% CI 3.8%, 12.3%).

### Relationships between War Trauma and EVD Exposure, Mental Health, and EVD-Related Health Behaviors

Initial analyses showed that when personal EVD exposures were considered together, only EVD diagnosis of a friend showed a significant relationship with individual EVD prevention or risk behaviors; the other EVD exposures were thus dropped from all models for parsimony.

Relationships between war exposures, personal EVD exposure, mental health, and EVD-related health behaviors are displayed in Table 3. EVD risk behaviors were positively associated with depression symptom severity (*b* = 0.05; 95% CI 0.00, 0.10; *p =* 0.037) and PTSD symptom severity (*b* = 0.10; 95% CI 0.03, 0.17; *p =* 0.008) and inversely associated with war exposures (*b* = −0.09; 95% CI −0.17, −0.02; *p =* 0.036) and having a friend diagnosed with EVD (*b* = −0.04; 95% CI −0.08, −0.00; *p =* 0.036). Both PTSD (*b* = 0.47; 95% CI 0.31, 0.63; *p <* 0.001) and depression (*b* = 0.51; 95% CI 0.26, 0.76; *p <* 0.003) were associated with war exposures. The unstandardized coefficients are in terms of their original units as displayed in Table 2; for example, having a friend who had EVD is associated with an overall decrease in the risk behavior score of 0.04, which is about 17.6% of a standard deviation of that score. A decrease in the average response to the severity of depression scale of roughly 0.55 (a little more than halfway between responses on the Likert scale)—which corresponds to one standard deviation—results in an approximate 0.03 change in the outcome, which amounts to a 11.5% of a standard deviation change. Considering all variables in the model predicting EVD risk behaviors displayed in Table 3 and the additional steps to assess mediation, war exposures have an indirect positive effect on EVD risk behaviors through PTSD symptoms and depression; however, accounting for this effect, there is a negative direct effect of war exposures (i.e., tending to reduce EVD risk behaviors). Because the direct and indirect effects are in opposite directions, they act against each other and thus the total effects are closer to zero and not significant. Table 4 displays the direct, indirect, and total effects for depression and PTSD as mediators of war exposure’s effect on EVD risk and prevention behaviors.

**Table 3:** Estimated coefficients predicting EVD prevention and risk behaviors.

Independent Variable	EVD Prevention Behaviors		EVD Risk Behaviors	
	Coefficient (95% CI)	*p-*Value	Coefficient (95% CI)	*p-*Value
Anxiety score	0.23 (0.06, 0.40)	0.008	0.03 (−0.04, 0.11)	0.381
Depression score	−0.15 (−0.38, 0.07)	0.180	0.05 (0.00, 0.10)	0.037
PTSD score	−0.24 (−0.43, −0.06)	0.009	0.10 (0.03, 0.17)	0.008
War exposures	0.45 (0.16, 0.74)	0.003	−0.09 (−0.17, −0.02)	0.013
Friend had EVD	0.15 (0.04, 0.27)	0.011	−0.04 (−0.08, −0.00)	0.036

**Table 4:** Decomposition of effects of war exposures on EVD risk and prevention behaviors.

Outcome	Direct Effect of War Exposure (95% CI)	Indirect Effect through PTSD (95% CI)	Indirect Effect through Depression (95% CI)	Total Effect (95% CI)
Risk behaviors	−0.09 (−0.17, −0.02)	0.02 (0.01, 0.08)	0.04 (0.00, 0.05)	−0.03 (−0.11, 0.06)
Prevention behaviors	0.45 (0.16, 0.74)	−0.07 (−0.19, −0.02)	−0.10 (−0.18, 0.04)	0.22 (−0.09, 0.53)

EVD prevention behaviors (Table 3) such as frequent hand washing and avoiding mass gatherings were associated with higher levels of anxiety (*b* = 0.23; 95% CI 0.06, 0.40; *p =* 0.008). Depression was not significantly associated with EVD prevention behaviors. Having a friend diagnosed with EVD (*b* = 0.15; 95% CI 0.04, 0.27; *p =* 0.011) and higher levels of war exposure (*b* = 0.45; 95% CI 0.16, 0.74; *p =* 0.003) were associated with greater EVD prevention behaviors, while PTSD symptoms were associated with fewer EVD prevention behaviors (*b* = −0.24; 95% CI −0.43, −0.06; *p =* 0.009). As above, the coefficients are in the original units of the scale. For example, having a friend diagnosed with EVD is related to an increase in the level of preventative behaviors of 0.15, which is about 24.6% of a standard deviation. A change in the response for anxiety symptoms, about one standard deviation, was related to an increase of about 0.13 on the preventative behavior scale, which is about 21.3% of a standard deviation. Similar to the effect seen above, war exposures had negative indirect effects on EVD prevention behaviors, but in the presence of mental health mediators, there was a positive direct effect of war exposures on preventative behaviors (Table 4).

## Discussion

Consistent with our conceptual model, higher scores on measures of PTSD symptoms and depression were associated with higher EVD risk behaviors, and symptoms of PTSD were associated with lower levels of EVD prevention behaviors. In other words, chronic mental health difficulties associated with war trauma were associated with higher levels of risk behaviors in the context of the EVD epidemic. Furthermore, we showed that both PTSD and depression were in part associated with exposure to traumatic events during Sierra Leone’s civil war.

For both EVD prevention and EVD risk behaviors, the behaviors of participants were associated with EVD diagnosis of a friend, more so than with diagnosis of EVD among neighbors or other community members, suggesting that intimate knowledge of affected patients may be more important than geographic proximity in shaping one’s personal response to the epidemic.

After accounting for the association between war exposures and adverse mental health consequences, higher levels of exposure to war-related events were associated with EVD prevention behaviors; such a relationship might be indicative of individuals with higher levels of war exposure being persons with stronger survival skills or who have become more risk adverse.

Our results are consistent with relationships established between depression and PTSD in research on HIV risk behaviors [15]. As has been found in HIV research, in the presence of common mental health problems such as depression, good judgment was possibly distorted, leading to our finding that higher levels of depression symptoms were associated with higher levels of EVD risk behaviors. Traumatic stress reactions may also play a similar role whereby, in the presence of PTSD symptoms, the ability of the individual to attend to EVD prevention activities was less. However, higher levels of anxiety symptoms were associated with more EVD prevention behaviors such as hand washing and seeking out EVD information via healthcare workers, suggesting that anxious persons may maintain a higher level of vigilance or concern about the EVD epidemic or that vigilance may have raised anxiety in some individuals who were more actively taking precautions. It is also difficult to disentangle the relationship of anxiety to the EVD epidemic. It is possible that an unmeasured trait, such as vigilance, underlies both anxiety and preventive behaviors.

### Study Limitations

Although this is, to our knowledge, the first study of its kind to examine the relationship between both past war exposure and mental health and present-day EVD-related health behaviors, limitations must be noted. Although we used randomly selected census EAs to derive a representative sample of households in the Western Area at the height of the epidemic, this sample is specific to Freetown and the Western Area and not representative of Sierra Leone’s other 13 districts. However, this sample is of relevance for understanding the dynamics related to EVD and mental health in other major capital cities in the EVD-affected countries of West Africa. The analyses may also be subject to some degree of confounding. In addition, the measures of our main outcomes as well as personal EVD exposure, war exposures, and mental health predictors rely on self-report, and therefore raise the possibility of common methods bias.

### Conclusion

In post-conflict settings, high levels of unaddressed mental health problems are common and are associated with war exposure. Such mental health difficulties can in turn shape the uptake of sensitization campaigns and public health messages aimed at reducing EVD risk. The findings in this study indicate the need for greater attention to the role of PTSD, depression, and other common mental health problems in counteracting risks for EVD in post-conflict Sierra Leone and may have implications for other war-affected regions. More generally, the findings suggest that successful uptake of EVD prevention messages among individuals with poor mental health in post-conflict low- and middle-income countries requires specific and targeted approaches that take into account the nature of war traumas, the resulting behavioral implications, and the potentially re-traumatizing effect of communication around fear and death, which are exacerbated in the context of such crises but remain underaddressed. The EVD epidemic laid bare the weaknesses in Sierra Leone’s health services. The country’s highly underdeveloped mental health and social services system must be strengthened to respond to the reality of compounded adversity due to both war and the recent epidemic and to prevent future outbreaks [37].

## Supporting Information

S1 STROBE Checklist (DOCX)

S1 Table EVD risk behavior scale. (DOCX)

S2 Table EVD prevention behavior scale. (DOCX)

S3 Table Mental health section, including PTSD scale, of study questionnaire. (DOCX)

## Abbreviations

EA: enumeration area
EVD: Ebola virus disease
HSCL-25: Hopkins Symptom Checklist-25
PTSD: post-traumatic stress disorder
