# Supplementary material for: Associations between Mental Health and Ebola-Related Health Behaviors: A Regionally Representative Cross-sectional Survey in Post-conflict Sierra Leone
Source: PLoS Med. 2016 Aug 9;13(8):e1002073. doi: 10.1371/journal.pmed.1002073 (PMC4978463; doi:10.1371/journal.pmed.1002073)
Supplement: S1 Table — (DOCX) [file pmed.1002073.s002.docx]

**S1 Table. EVD risk behavior scale.**

| **Risk behaviors - 14 items** |
| --- |
| *If someone in your household were to become sick with symptoms such as fever, fatigue, malaise and weakness, reddened eyes, joint and muscle pain, headache, nausea and vomiting, how likely would you do the following?* |
| Wait and see if symptoms got worse |
| Wait and see if the symptoms would go away |
| Keep them at home and provide care |
| Get help from my family at home |
| Move them to another location that is not an Ebola treatment center where I know that family or friends who I know would take care of them |
| Consult a traditional healer |
| Make them take a hot salt water bath |
| *If YOU were to become sick with symptoms such as fever, fatigue, malaise and weakness, reddened eyes, joint and muscle pain, headache, nausea and vomiting, how likely would you do the following?* |
| Wait and see if symptoms got worse |
| Wait and see if the symptoms would go away |
| Stay home and provide my own care |
| Get help from my family at home |
| Move to another location that is not an Ebola treatment center where I know that family or friends who I know would take care of me |
| Consult a traditional healer |
| Take a hot salt water bath |
