# Supplementary material for: Associations between Mental Health and Ebola-Related Health Behaviors: A Regionally Representative Cross-sectional Survey in Post-conflict Sierra Leone
Source: PLoS Med. 2016 Aug 9;13(8):e1002073. doi: 10.1371/journal.pmed.1002073 (PMC4978463; doi:10.1371/journal.pmed.1002073)
Supplement: S2 Table — (DOCX) [file pmed.1002073.s003.docx]

**S2 Table. EVD prevention behavior scale.**

| **Prevention behaviors - 16 items** |
| --- |
| *As a result of Ebola, have you done any of the following?* |
| Avoided places where many people are gathered together, like sporting events, markets, or public transportation |
| Reduced human contact with people inside of your immediate family such as signs of affection like hug, kiss or shaking hands |
| Reduced human contact such as signs of affection such as hug/kiss or shaking hands with people outside of your immediate family |
| Talked with a community leader about issues related to Ebola |
| Talked with a health worker about issues related to Ebola |
| Bought a face mask |
| Avoided people you think may have recently visited an Ebola-affected area |
| Avoided someone known to be sick with Ebola |
| Avoided the body of someone who recently died of Ebola |
| Avoided people you think may have recently visited someone with Ebola |
| Gotten a prescription for or purchased antibiotics such as Cipro |
| Avoided mass transit such as buses |
| Avoided public transit such as local taxis |
| Avoided public transit such as local motorbikes |
| Avoided visiting relatives |
| Avoided visiting neighbors |
