# Supplementary material for: Associations between Mental Health and Ebola-Related Health Behaviors: A Regionally Representative Cross-sectional Survey in Post-conflict Sierra Leone
Source: PLoS Med. 2016 Aug 9;13(8):e1002073. doi: 10.1371/journal.pmed.1002073 (PMC4978463; doi:10.1371/journal.pmed.1002073)
Supplement: S3 Table — (DOCX) [file pmed.1002073.s004.docx]

**S3 Table. Mental health section, including PTSD scale, of study questionnaire.**

| Now I am going to ask you some questions about the most distressing event that you have experienced, witnessed, or were confronted with which caused intense fear, helplessness, or horror. If you feel able, can you tell me what that event was? _________________ |
| --- |
| Was the distressing event you described above war-related? |
|  |
| **PTSD Symptom Scale Interview - 16 items** |
| *In the past six months…* |
| **Re-experiencing** |
| Have you had recurrent or intrusive distressing thoughts or recollections about the trauma? |
| Have you been having recurrent bad dreams or nightmares about the trauma? |
| Have you had the experience of suddenly reliving the trauma, flashbacks of it, acting or feeling as if it were re-occurring? |
| Have you been intensely EMOTIONALLY upset when reminded of the trauma includes anniversary reactions)? |
| Have you been having intense PHYSICAL reactions like sweaty, heart palpitations or other things that happen when reminded of the trauma? |
| **Avoidance** |
| Have you persistently been making efforts to avoid thoughts or feelings associated with the trauma? |
| ***Have you persistently been making efforts to avoid activities, situations, or places that remind you of the trauma? (omitted)*** |
| Are there any important aspects about the trauma that you still cannot recall? |
| Have you markedly lost interest in free time activities since the trauma? |
| Have you felt detached or cut off from others around you since the trauma? |
| Have you felt that your ability to experience the whole range of emotions is impaired such as (i.e. unable to have loving feelings)? |
| Have you felt that any future plans or hopes have changed because of the trauma for example no career, marriage, children, or long life? |
| **Increased arousal** |
| Have you had persistent difficulty falling or staying asleep? |
| Have you been continuously irritable or have outbursts of anger? |
| Have you had persistent difficulty concentrating? |
| Are you overly alert for example check to see who is around you since the trauma? |
| Have you been jumpier, more easily startled, since the trauma? |
